# Conformational switches that control the TEC kinase – PLCγ signaling axis

**DOI:** 10.1016/j.yjsbx.2022.100061

**Published:** 2022-01-22

**Authors:** Jacques Lowe, Raji E. Joseph, Amy H. Andreotti

**Affiliations:** Roy J. Carver Department of Biochemistry, Biophysics and Molecular Biology, Iowa State University, Ames, IA 50011, USA

**Keywords:** BTK, ITK, SH3, SH2, PHTH, PRR, TEC, PLCγ, γSA, sPH, DAG, IP_3_, PIP_3_, IP_4_, SAXS, NMR

## Abstract

Cell surface receptors such as the T-cell receptor (TCR) and B-cell receptor (BCR) engage with external stimuli to transmit information into the cell and initiate a cascade of signaling events that lead to gene expression that drives the immune response. At the heart of controlling T- and B-cell cell signaling, phospholipase Cγ hydrolyzes membrane associated PIP_2_, leading to generation of the second messengers IP_3_ and DAG. These small molecules trigger mobilization of intracellular Ca^2+^ and promote transcription factor transport into the nucleus launching the adaptive immune response. The TEC family kinases are responsible for phosphorylating and activating PLCγ, and our group aims to understand mechanisms that regulate immune cell signal transduction by focusing on this kinase/phospholipase axis in T-cells and B-cells. Here, we review the current molecular level understanding of how the TEC kinases (ITK and BTK) and PLCγ1/2 are autoinhibited prior to activation of cell surface receptors, how TEC kinases are activated to specifically recognize the PLCγ substrate, and how conformational changes induced by phosphorylation trigger PLCγ activation.

## Highlights

• TEC kinases and PLCγ transition between autoinhibited state and active conformation.
• PLCγ structures reveal both autoinhibited form and active form of gamma specific array (γSA); the four regulatory domains unique to the PLCγ isozymes.
• Domain dynamics likely control activation mechanism.
• PLCγ phosphorylation triggers conformational switch.

## Graphical abstract

**Figure:**
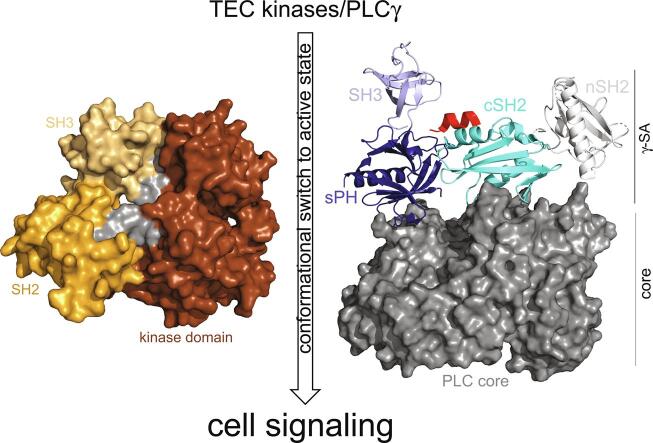

## Introduction

### TEC kinases and phospholipases (Fig. 1)

**Fig. 1:**
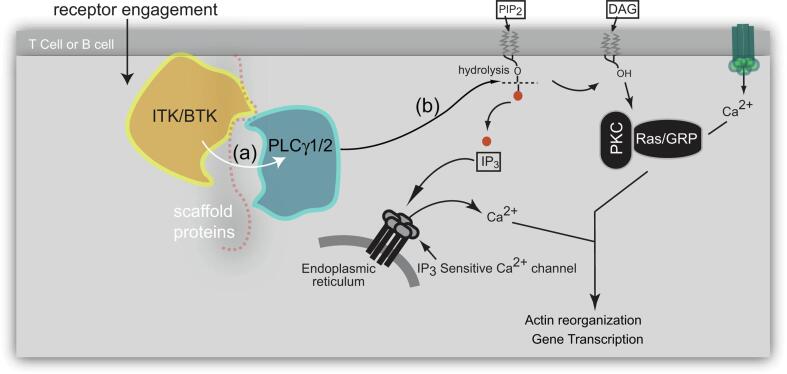
**Signal transduction through the TEC family kinases and PLCγ enzymes.** Antigen recognition by T- and B-cell receptors initiates a cascade of signaling events, ultimately resulting in actin reorganization and specific expression of genes coding for cytokines, antibodies, and other effectors. Signaling proteins proximal to the antigen receptor assemble on membrane associated scaffolding proteins and drive production of second messengers that are essential for orchestrating the spatial and temporal flow of signal transduction. The TEC kinases (ITK or BTK) phosphorylate and activate phospholipase C gamma 1/2 (PLCγ1/2); a key signaling enzyme that produces the second messengers inositol 1,4,5-trisphosphate (IP_3_) and diacylglycerol (DAG) from hydrolysis of phosphatidylinositol 4,5-bisphosphate (PIP_2_) at the membrane. IP_3_ and DAG generate an influx of intracellular Ca^2+^ and activate the Ras/MAPK pathways, respectively leading to actin reorganization and activation of transcription factors such as NFAT, NF-κB and AP-1.

Tyrosine kinases are first responders that propagate cellular signals immediately following membrane receptor engagement. In a subset of immune cells including T- and B-cells, the TEC family kinases are the third in a series of proximal non-receptor tyrosine kinases that are activated by phosphorylation. Activated TEC family kinases ITK and BTK are assembled within the membrane associated scaffolding protein complex and phosphorylate their substrate phospholipase Cγ (PLCγ) (Fig. 1**a**). In the absence of activating stimuli, TEC kinases adopt an autoinhibited conformation that limits unwarranted catalytic activity. TEC family kinases have been notoriously resistant to crystallizing in their full-length form leaving open many questions regarding the molecular details of regulation.

PLC’s are a central node in controlling many aspects of cell signaling and gene expression. The PLCγ subtype consists of PLCγ1 and PLCγ2 isoforms that are expressed in T-cells and B-cells respectively among other cell types. Activated PLCγ1/2 hydrolyzes phosphatidylinositol 4,5-bisphosphate (PIP_2_) to generate the second messengers inositol trisphosphate (IP_3_) and diacylglycerol (DAG) (Fig. 1**b**) triggering further signaling events that ultimately result in changes in gene transcription, that for T- and B-cells, result in activation of the adaptive immune response.

This graphical review directs its attention to the conformational changes and molecular binding events that control the transition between autoinhibited and activated TEC kinases and phospholipase Cγ. Throughout, we highlight available data to build a structural understanding of the molecular events that regulate the TEC kinases and the phospholipase C gamma family; sequence and structural homology between BTK and ITK, as well as PLCγ1 and PLCγ2, suggest that structural features determined for one family member are likely shared across the protein family.

## Tec family kinase autoinhibition (Fig. 2)

Non-receptor tyrosine kinases (NRTKs) are cytosolic enzymes that are activated following receptor engagement. The well-studied SRC family kinases are the prototypical example of NRTKs and make up the largest family of NRTKs with 9 distinct kinases. The SRC kinase sequences all include a conserved C-terminal regulatory tail that contains an inhibitory tyrosine phosphorylation site (Y527 in SRC). The precise regulatory role of SRC Y527 became clear when the first SRC kinase was crystalized in 1997 (Xu et al., 1997). Phosphorylated Y527 (pY527) interacts with the SH2 domain in the same molecule stabilizing the compact autoinhibitory state. Loss of the Y527 hydroxyl (mutation of Y527 to phenylalanine) results in increased propensity for the SRC kinase to adopt an open, active conformation.

**Fig. 2:**
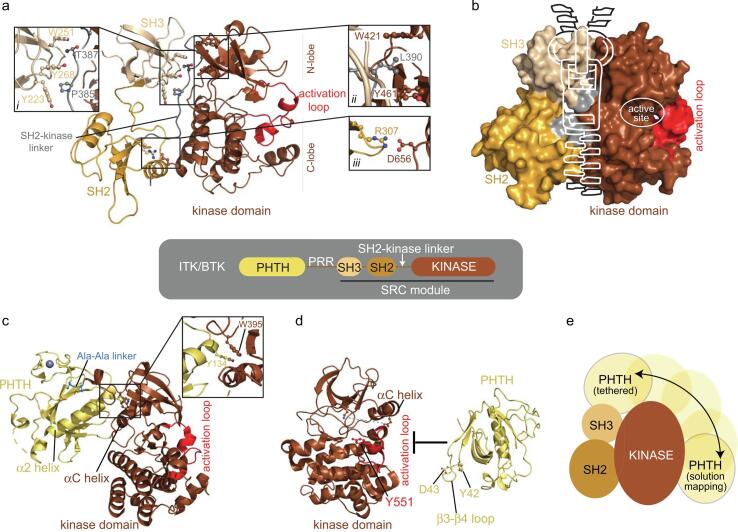
**Domain structure and autoinhibited pose of ITK/BTK.** The TEC kinases all contain the architectural core defined as the SRC-module comprised of a SRC-Homology 3 (SH3), SRC-Homology 2 (SH2), and kinase domain (KD) (**center**). (**a,b**) The crystal structure (PDB ID: 4XI2) of the BTK SH3-SH2-kinase fragment depicts the SH3 (tan) and SH2 (orange) domains contacting the distal surface of the kinase domain (brown), opposite the activation loop, which is collapsed into the catalytic cleft (Wang et al., 2015). Residues 383–387 from the SH2-kinase linker form a polyproline type-ll helix that is sandwiched between the SH3 peptide-binding pocket and the N-lobe of the kinase domain (**a,*i***). In addition, this region contains the ‘hydrophobic stack’ (von Raussendorf et al., 2017) that is comprised of L390 in BTK (ITK^L351^) from the SH2-kinase linker inserted between two hydrophobic residues W421 (ITK^W382^) and Y461 (ITK^Y422^) that protrude from the back of the kinase domain N-lobe (**a, *ii***). The hydrophobic stack not only stabilizes the autoinhibited structure, but it also allosterically communicates with the active site (circled in b). The SH2 domain takes up its inhibitory position through contacts with the C-lobe of the kinase domain. A salt bridge between the side chain of R307 (ITK^R265^) in the SH2 domain and D656 (ITK^E616^) at the C-terminus of the kinase domain (**a, *iii***) maintains the autoinhibited conformation and loss of the acidic side chain leads to kinase activation (Joseph et al., 2017). Thus, while not subject to post-translational control, D656 in BTK mimics the role of SRC pY527. (**b**)The interdomain contacts between the SH3-SH2, SH2-kinase linker and Kinase domain “zip” the molecule into the autoinhibitory structure. Crystal structure of the PHTH domain (gold) tethered directly to the kinase domain via a Ala-Ala dipeptide (**c**, PDB ID: 4Y93) reveals a contact between the PHTH α2 helix and N-lobe of the kinase domain (Wang et al., 2015). W395 at the start of the kinase domain and the nearby αC helix in the kinase domain N-lobe create a groove that accommodates Y134 from the terminus of the PHTH α2 helix. Alternative autoinhibitory position for the PHTH domain (**d**) characterized based on solution-based approaches. Residues 42 and 43 on the β3-β4 loop of the PHTH domain mediates an autoinhibitory contact with the catalytic face of the C-lobe kinase domain, reducing accessibility of the activation loop (as measured by accessibility of Y551) and stabilizing the autoinhibited state of the kinase domain (αC helix ‘out’ disrupting the Lys/Glu salt bridge (broken grey line) of the active state). Unlike the well-defined arrangement of the SH3-SH2-kinase region of the autoinhibited TEC kinases, the PHTH domain may adopt multiple conformations in the autoinhibited enzyme (**e**). Such a model would be consistent with the PHTH domain performing a surveillance role, scanning the membrane for the presence of its ligand, phosphatidylinositol (3,4,5)-trisphosphate (PIP_3_). Indeed, soluble Inositol 1,3,4,5-tetrakisphosphate (IP_4_) does not activate BTK in vitro (Wang et al., 2015) suggesting that, unlike ligand binding to the BTK SH3 and SH2 domains, IP_4_ binding alone is insufficient to shift the conformational preference away from the autoinhibited state. Interestingly, there is some data supporting a cellular role for IP_4_ activation of ITK (Huang et al., 2007) but the precise mechanism by which this occurs is not clear. (For interpretation of the references to color in this figure legend, the reader is referred to the web version of this article.)

The second largest family of NRTKs are the TEC kinases. The five members of the TEC family (ITK, BTK, TXK (RLK), TEC & BMX) are expressed primarily in cells of hematopoietic lineage and none of the TEC kinases contain the C-terminal regulatory phosphorylation site that is so critical in controlling the function of the SRC family kinases. To date, a crystal structure of any full-length autoinhibited TEC kinase is lacking but in 2015, a fragment containing just three of the domains in the TEC family kinase BTK was crystallized (Wang et al., 2015) representing the first glimpse into how the TEC kinase domains are arranged in three-dimensional space (Fig. 2**a,b**). The SRC-module (SH3-SH2-kinase fragment) crystallized as a domain swapped dimer with domain swapping occurring in the SH2 domain. While the functional significance of the domain swapped dimer is unknown, each half of the domain swapped dimer adopts an autoinhibitory conformation quite similar to that observed for the SRC family. The SH3 and SH2 domains assemble on the ‘back’ of the kinase domain with the SH2-linker region contacting both the SH3 and kinase domain N-lobe (Fig. 2**a,*i,ii***). This domain arrangement is similar to SRC kinases despite the absence of the SRC regulatory tail in the TEC family. Solution studies of BTK (Joseph et al., 2017), revealed the negatively charged side-chain D656 fills the role of SRC pY527 by mediating inhibitory contacts between the end of the kinase domain and the BTK SH2 domain (Fig. 2**a,*iii***). In addition, interactions between the SH2-kinase linker and the N-lobe of the kinase assemble a hydrophobic stack (Fig. 2**a, *ii***) which stabilizes the autoinhibited structure and also allosterically communicates with the active site; formation of the inhibitory hydrophobic stack leads to selective binding of ADP over ATP in the kinase domain, an observation first established with ITK and SRC (von Raussendorf et al., 2017). This work provided a clear link between the regulatory interactions of the SH3/SH2-linker region and the catalytic function of the active site (von Raussendorf et al., 2017).

A distinguishing feature of the TEC family is the Pleckstrin-Homology Tec-Homology (PHTH) domain followed by a long linker region that contains a proline-rich region (PRR) that matches the canonical SH3 ligand sequence (Fig. 2**, center**). Accurately defining the autoinhibitory contacts for the PHTH domain and the role of the PRR has been challenging (Wang et al., 2015, Amatya et al., 2019). A crystal structure of the PHTH domain tethered directly to the kinase domain via a Ala-Ala dipeptide has been solved (Fig. 2**c**) and reveals a contact between the PHTH α2 helix and N-lobe of the kinase domain (Wang et al., 2015). The position of the PHTH domain in the tethered crystal structure (Fig. 2**c**) partially overlaps with the SH3 domain in the structure of the autoinhibited SRC module of BTK (Fig. 2**a**) and so these structures are mutually exclusive. It is possible that this binding mode represents an intermediate on the activation pathway instead of a stable autoinhibited structure. An alternative autoinhibitory position for the PHTH domain (Fig. 2**d**) has been characterized based on nuclear magnetic resonance (NMR) data and hydrogen–deuterium exchange applied to the full-length BTK molecule (Amatya et al., 2019, Devkota et al., 2017). These solution-based approaches suggest that the β3-β4 loop of the PHTH domain mediates an autoinhibitory contact with the catalytic face of the C-lobe kinase domain stabilizing the autoinhibited state of the kinase domain. Given the multiple interdomain contacts observed between the PHTH and kinase domain (Fig. 2**c,d**) it is likely that the PHTH domain is dynamic and possibly sampling a range of conformational states with respect to the autoinhibited SRC module (Fig. 2**e**).

Current models of autoinhibited full-length BTK do not resolve the proline-rich region (PRR). Available data suggest that this long linear motif situated between PHTH and SH3 domains (Fig. 2**, center**) might serve to ‘prime’ the full-length kinase for activation (Devkota et al., 2015). The canonical SH3 ligand sequence within the PRR binds to the adjacent SH3 domain in a manner that competes with the autoinhibitory contact between the SH3 binding groove and the SH2-kinase linker. Mutation of the PRR results in a more stable autoinhibited state and lower catalytic activity in vitro (Joseph et al., 2017). It is also likely that the PRR of the TEC kinases serves as a docking site for other SH3 containing signaling proteins during signaling; in fact, the BTK PRR contains two consecutive SH3 ligands setting it apart from the other kinases in the family.

## PLCγ autoinhibition (Fig. 3)

Studies have shown that mutants of both PLCγ1 and PLCγ2 have been implicated in driving human diseases such as leukemias and lymphomas, as well as being associated with dysregulated immune responses and resistance to inhibitors. Therefore, numerous experimental approaches have been carried out to structurally characterize the PLCγ isozyme (Bunney et al., 2012, Hajicek et al., 2019).

**Fig. 3:**
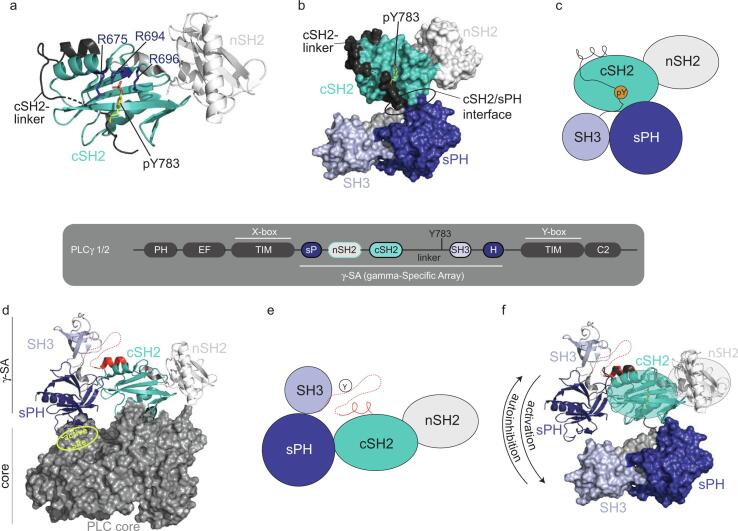
**The gamma subfamily of PLC’s contain a unique regulatory unit; the gamma-Specific Array (γSA).** PLC isozymes share a common core that includes: a PH domain, two pairs of EF hands, a catalytic TIM barrel and a C2 domain. The γSA consists of a split PH domain (sPH), two SH2 domains (nSH2 and cSH2), a long linker region that contains the regulatory phosphorylation site (Y783 in PLCγ1), and an SH3 domain (**center**). The γSA divides the catalytic TIM barrel into its respective X- and Y- boxes. (**a**) Structure (PDB ID: 4EY0) of phosphorylated nSH2-cSH2-linker reveals a canonical pY/SH2 interaction between pY783 and the arginine lined binding pocket of cSH2 (R675, 694 & 696, blue sticks). The cSH2 binding pocket also showed electron density for the structure of unphosphorylated PLCγ1 nSH2-cSH2-linker suggesting that the Y783 containing linker binds to the cSH2 domain even in the absence of phosphorylation. (**b**) Structural model of the γSA using a combination of NMR and small angle X-ray scattering (SAXS). In this model, the sPH-SH3 segment contacts the cSH2 domain (via an sPH/cSH2 interface) involving the βD-βE strands of the cSH2 domain (**c**) Cartoon representing the relative orientation of the domains shown in (**b**). (**d**) Crystal structure (PDB ID: 6PBC) of full-length PLCγ1 shows that the sPH (dark blue) and cSH2 (teal) domains both form extensive autoinhibitory contacts with the core of PLCγ1 (shown in grey surface rendering) thereby blocking membrane engagement and access to the PIP_2_ substrate. The cSH2-linker region of PLCγ1 (indicated by dotted red line in (**d**)) is not evident in the full-length crystal structure. The cSH2/core interface is coincident with the binding site of the phosphorylated Y783 linker (shown in (**a**)) suggesting that release of the cSH2 domain from the core is triggered upon phosphorylation. (**e**) Cartoon representing the relative orientation of the γSA domains shown in (**d**). (**f**) Superposition of the nSH2-cSH2 region from the γSA structure derived from SAXS (**b**) and that extracted from the full-length structure (**d**) show that the sPH-SH3 unit occupies completely different arrangements with respect to nSH2-cSH2 in the two models. sPH/SH3 is shown as surface rendered for early SAXS derived model and as ribbons for arrangement revealed by crystallography of full-length autoinhibited PLCγ. The cSH2-linker region remains undefined (and likely flexible) in the autoinhibited form (red) while in the active form (phosphorylated on Y783, shown in gray as in (**a**)), the cSH2-linker contacts the cSH2 domain in an intramolecular manner. These observations suggest that the earlier models of γSA (**b,c**) represent an active form of PLCγ instead of the inactive state suggested at the time (Joseph et al., 2021). A large domain rearrangement is required to transition to the autoinhibited state; the sPH-SH3 segment rotates up and out to adopt the autoinhibited form (**d,e**). This arrangement reveals the sPH/cSH2 surface required for capping of the PLC core by the γSA (**d**). (For interpretation of the references to color in this figure legend, the reader is referred to the web version of this article.)

Some of the first structural work on PLCγ involved determination of two crystal structures for the tandem nSH2-cSH2-linker region; one phosphorylated on Y783 and the other without phosphorylation (Bunney et al., 2012). The structure of phosphorylated nSH2-cSH2-linker reveals a canonical pY/SH2 interaction between pY783 and the arginine lined binding pocket of cSH2 (Fig. 3**a**). The cSH2 binding pocket also showed electron density for the structure of unphosphorylated PLCγ1 nSH2-cSH2-linker, which the authors suggest that the Y783 containing linker binds to the cSH2 domain even in the absence of phosphorylation. Using a combination of NMR and small angle X-ray scattering (SAXS), the same authors produced a structural model of the entire γSA. In this model, the sPH-SH3 segment contacts the cSH2 domain (via an sPH/cSH2 interface) involving the βD-βE strands of the cSH2 domain (Fig. 3**b,c**). Split PH domains are characterized by insertions of one or more autonomously folded protein modules in the middle of pH domain sequences, which is an uncommon feature for PH domains making split PH domains quite unique (Wen et al., 2006).

Advances in cryoEM and success in crystallizing the full-length PLCγ1 enzyme (Hajicek et al., 2019, Liu et al., 2020) yielded the first glimpse of the complete autoinhibitory structure of this class of phospholipase. The autoinhibited structure shows that the sPH and cSH2 domains both form extensive autoinhibitory contacts with the core of PLCγ1 (Fig. 3**d,e**). These inhibitory interfaces latch the γSA to the top of the catalytic core and block access to the PIP_2_ substrate. The cSH2-linker region of PLCγ1 is not evident in the full-length crystal structure and was in fact shortened considerably in order to obtain crystals of the full-length PLCγ1. The cSH2/core interface is coincident with the binding site of the phosphorylated Y783 linker suggesting that release of the cSH2 domain from the core is triggered upon phosphorylation. In addition, the association of the non-phosphorylated Y783 containing linker with cSH2 (Bunney et al., 2012) suggests that autoinhibited PLCγ1 samples an open conformation prior to Y783 phosphorylation-induced activation. In fact, any mechanism that competes with the stable, latched γSA/core interaction will shift the equilibrium away from the autoinhibited form and result in increased accessibility to PIP_2_ substrate and thus increased activity of the enzyme. For example, mutations in PLCγ2 drive drug resistance in part by shifting the optimal equilibrium away from autoinhibited enzyme (Joseph et al., 2021).

Comparison of the two available structural models for the PLCγ1 γSA region (Fig. 3**f**) show two distinct arrangements of the sPH/SH3 cassette with respect to the nSH2-cSH2 segment. It is suggested that a large conformational change occurs in the γSA region upon PLCγ1 activation (Gresset et al., 2010) and so the conformational states revealed to date by structural biology approaches may represent the autoinhibited form (as confirmed by the full-length structure, Fig. 3**d**) and the putative active state (captured by solution methods focused on the isolated γSA region, Fig. 3**b**). In essence, the models suggest that activation via phosphorylation of Y783 competes with the cSH2/core interaction allowing the sPH/SH3 domains to swing down and interact with a different surface on cSH2. The conformational adjustment initiated by pY783 binding to the cSH2 domain would result in release of γSA from the PLCγ core followed by steric occlusion of the autoinhibitory surface of γSA. This activation mechanism may, in part, serve to prevent re-association of cSH2 with the PLCγ core to maintain the active state of PLCγ as it encounters its substrate at the membrane. Additional experiments are needed to test this idea and gain a deeper appreciation for the dynamics that control activation/autoinhibition of the PLCγ family.

**Fig. 4:**
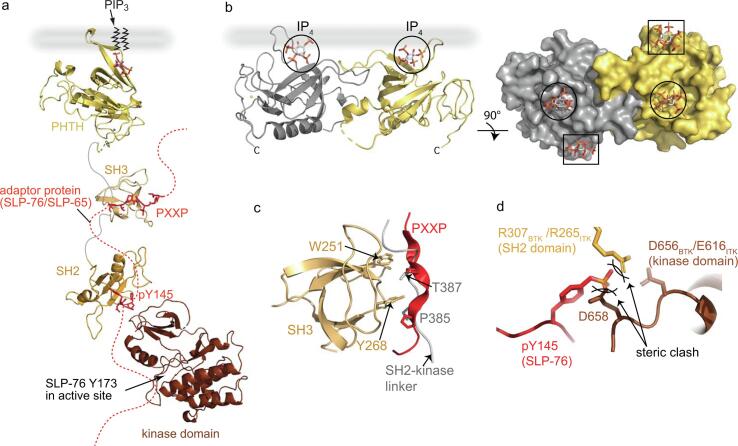
**Activation of the TEC kinases.** T-cell or B-cell stimulation generates ligands for the regulatory domains of the TEC kinases which promote the formation of signaling complexes that nucleate around non-catalytic scaffolding proteins such as SLP-76 in T-cells (or SLP-65 in B cells). These interactions with the regulatory domains disrupt the ‘closed’ autoinhibited conformation of TEC kinases to a more ‘open’ form (**a**). Crystal structures reveal dimerization (**b, left**) of the BTK N-terminal PHTH domain bound to the PIP_3_ headgroup, IP_4_, now known as the ‘Saraste dimer’. Subsequent structures of the BTK PHTH dimer (Wang et al., 2015) revealed additional phospholipid binding sites; the canonical site (**circled**) as well as a peripheral site (**boxed**) on each domain in the dimer (**b, right**). Elegant studies using fluorescence correlation spectroscopy and membrane-binding kinetic measurements revealed a role for the peripheral site in setting a PIP_3_ threshold required to trigger BTK activation (Chung et al., 2019). (**c**) Interactions between the PXXP motif (red) in adaptor proteins and the SH3 domains of ITK/BTK (Bunnell et al., 2000) contribute to the unraveling of the compact autoinhibitory pose by displacing the SH2-kinase linker (grey). (**d**) Phosphotyrosine binding (SLP-76 pY145 is shown in (**a**)) to the ITK/BTK SH2 domain is sterically incompatible with the autoinhibitory interaction between the SH2 and the C-terminal region of the kinase domain (shown in Fig. 2**a,*iii***) leading to displacement of the intramolecular SH2 inhibitory interaction and a shift toward the active conformation. (For interpretation of the references to color in this figure legend, the reader is referred to the web version of this article.)

**Fig. 5:**
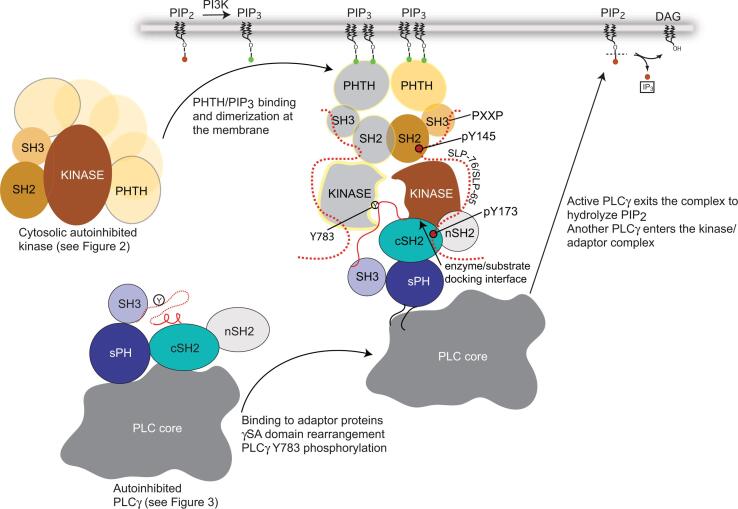
**Activation and phosphorylation of PLCγ by TEC kinases.** The TEC kinase/PLCγ enzyme substrate pair meet within a larger signaling complex that involves the adaptor proteins SLP-76 and LAT in T-cells (SLP-65/BLNK in B cells is analogous to the SLP-76 adaptor protein). Receptor activation leads to the activation of the lipid kinase, Phosphoinositide 3-kinase (PI3K) which produces membrane associated PIP_3._ Binding of PIP_3_ by the BTK PHTH domain leads to membrane recruitment and dimerization. Additionally, receptor activation leads to the phosphorylation of adaptor/scaffold proteins on key tyrosines. Specifically, three tyrosines in the SLP-76 N-terminal acidic domain are phosphorylated by ZAP-70, and one of these post-translational modifications (SLP-76 pY145) recruits ITK into the scaffolded complex. The idea that activation via membrane association (as well as ligand binding to SH3 and SH2 domains) leads to a more extended conformation is supported by early SAXS data acquired for full-length BTK (Marquez et al., 2003). The model that emerged from scattering data indicates an elongated shape, consistent with each domain engaged with its cognate ligand. Notably, the Saraste dimer (Fig. 4b) is arranged such that the C-terminus of each PHTH domain is pointing away from the membrane binding face meaning the elongated BTK molecule could form dimers consistent with the SAXS model (one BTK molecule follows the color scheme in Fig. 2 and the other is shown in gray). The extent to which dimerization (or even higher order oligomerization) is also mediated by the other domains in BTK remains unknown but it is intriguing to note that the regulatory domains of both BTK and ITK form a variety of specific dimer interfaces (Brazin et al., 2000, Laederach et al., 2002). Full kinase activation requires phosphorylation of the conserved activation loop tyrosine (ITK Y511/BTK Y551, not shown for clarity) which is phosphorylated either by an upstream SRC family kinase or by autophosphorylation promoted by co-localized BTK at the membrane. Additional interactions between the SRC homology domains of PLCγ and scaffold proteins (for example SLP-76 pY173 interacts with PLCγ cSH2 (Devkota et al., 2015, Sela et al., 2011) as well as a specific enzyme/substrate docking interaction between the PLCγ cSH2 domain and the C-lobe of the ITK/BTK kinase assemble the enzyme substrate pair (Min et al., 2009). Docking of PLCγ cSH2 on the kinase domain not only brings Y783^PLCγ1^/Y759^PLCγ2^ into proximity of the kinase active site but also destabilizes the autoinhibited form as cSH2 residues that are normally buried in autoinhibited PLCγ are required for kinase docking. Once PLCγ is phosphorylated, pY783 can bind intramolecularly to the cSH2 domain and PLCγ is released from the kinase/scaffold complex allowing another molecule of PLCγ to enter the kinase/scaffold complex (Cruz-Orcutt et al., 2014) and undergo phosphorylation induced activation.

Adaptor proteins/ membrane association/substrate priming and docking (Fig. 4, Fig. 5)

Interactions of the regulatory domains of TEC kinases with their ligands leads to activation and recruitment to the receptor signaling complex (Fig. 4**a**). As depicted in Fig. 2e, the PHTH domain may be visiting multiple isoenergetic conformational states in the autoinhibited kinase. Crystal structures, biophysical approaches and molecular dynamic simulations of the BTK PHTH domain bound to PIP_3_, the headgroup of PIP_3_, IP_4_ or the soluble phosphoinositol IP_6_ (Wang et al., 2015, Wang et al., 2019, Chung et al., 2019), show that PHTH dimerization can occur and that the BTK PHTH domain harbors two sites for membrane interaction (Fig. 4**b**). BTK dimerization and the presence of multiple ligand binding sites may confer a switch-like activation of BTK (Chung et al., 2019) by requiring a threshold concentration of PIP_3_ to generate membrane associated active BTK. Binding of the BTK PHTH domain to membrane associated PIP_3_ also promotes conformational changes throughout the full-length BTK molecule. Hydrogen deuterium exchange mass spectrometry reveals increased exposure to solvent in all domains of BTK upon association with PIP_3_ containing liposomes (Joseph et al., 2017). The active conformation of BTK at the membrane has not been experimentally determined but early SAXS analysis (Marquez et al., 2003) revealed an elongated conformation that would be compatible with the PHTH dimer at the membrane and binding of the SH3 and SH2 domains to adaptor protein sequences (Fig. 4**a, 5**).

SH3 ligands (PXXP motifs) within the adaptor proteins can displace the SH2-kinase linker bound to the SH3 domain in the autoinhibited conformation stabilizing the active kinase within the signaling complex (Fig. 4**a,c**). Receptor activation also leads to phosphorylation of adaptor proteins such as SLP-76 (pY145) which recruits ITK into the scaffolded complex via a canonical ITK SH2/pY145 interaction that disrupts the autoinhibitory interaction between SH2 and kinase domain (Fig. 4**a,d**).

Interestingly, BTK is the target of Ibrutinib and other second-generation inhibitors used in the treatment of B-cell chronic lymphocytic leukemia (CLL). BTK is prone to mutations that elicit drug resistance and in at least one case the mutation shifts the conformational equilibrium toward the active state of the kinase thereby increasing cellular BTK activity promoting escape from drug induced inhibition (Joseph et al., 2020). As well, different active site inhibitors of BTK show markedly different effects on the conformational equilibrium of the full-length enzyme; Ibrutinib destabilizes the compact autoinhibited form of BTK while GDC0853 has no effect on the conformational preferences of BTK outside of the kinase domain (Joseph et al., 2020). The extent to which drug induced changes in the conformational equilibrium of BTK affect signaling processes is not yet known.

Once activated, BTK/ITK is assembled within the membrane associated adaptor protein complex. Into this environment enters PLCγ1/2, which exits its autoinhibited conformation to associate with the kinase domain and specific regions of adaptor/scaffold proteins (Fig. 5). Here, TEC family kinase mediated activation of PLCγ occurs and active PLCγ promotes hydrolysis of PIP_2_ (Fig. 5). The next stage of signaling can commence: action of PIP_2_ derived second messengers IP_3_ and DAG on calcium channels and the PKC/Ras pathway drive changes in cell morphology and gene transcription ultimately generating the adaptive immune response.

## Declaration of Competing Interest

The authors declare that they have no known competing financial interests or personal relationships that could have appeared to influence the work reported in this paper.
